# Direct antiviral agents for hepatitis C and drug interaction risk: A retrospective cohort study with real and simulated data on medication interaction, prevalence of comorbidities and comedications

**DOI:** 10.1371/journal.pone.0245767

**Published:** 2021-02-12

**Authors:** Raquel Boff da Costa, Marisa Boff Costa, Larisse Longo, Daniela Elisa Miotto, Gustavo Hirata Dellavia, Matheus Trucollo Michalczuk, Mario Reis Álvares-da-Silva

**Affiliations:** 1 Graduate Program Sciences in Gastroenterology and Hepatology, School of Medicine, Universidade Federal do Rio Grande do Sul (UFRGS), Porto Alegre, Brazil; 2 Experimental Gastroenterology and Hepatology Laboratory, Center for Experimental Research, HCPA, Porto Alegre, Brazil; 3 Gastroenterology Division, Hospital de Clínicas de Porto Alegre (HCPA), Porto Alegre, Brazil; Zagazig University, EGYPT

## Abstract

**Introduction and aim:**

Comorbidities and comedication are common in patients with hepatitis C, which could result in a risk of drug-drug interaction. The objective of this study was to evaluate the prevalence of comorbidities, comedication and drug-drug interactions involving direct-acting antivirals in this population.

**Methods:**

Comorbidities and comedications were evaluated in a retrospective cohort of hepatitis C patients. Drug-drug interactions were estimated in real life and with simulated data on comedications following drug regimens: telaprevir; elbasvir/grazoprevir, ombitasvir/paritaprevir/r/ritonavir (2D regimen), and sofosbuvir/simeprevir, sofosbuvir/daclatasvir, sofosbuvir/ledipasvir; 2D/dasabuvir (3D regimen); glecaprevir/pibrentasvir and sofosbuvir/velpatasvir/voxilaprevir. The interactions were evaluated according to the University of Liverpool database. Statistical analysis was performed by SPSS^®^ 18.0.

**Results:**

Data from 1433 patients with hepatitis C were evaluated. The mean patient age was 51.7 years (SD ± 10.7), and 50.6% were female. Direct-acting antivirals were prescribed for 345 (24.1%) patients, and a sustained virological response occurred in 264 (76.5%). The main comorbidities were systemic arterial hypertension [436 (30.4%)], diabetes mellitus [352 (24.6%)] and depression [130 (9.1%)]. The mean number of comorbidities was 1.52 (median [IQR] of 1.00 [1.00–2.00]). The mean number of comedications was 3.16 (median [IQR] of 3.00 [1.00–5.00]). A total of 12916 drug-drug interactions were found, of which 1.859 (14.4%) were high risk, with a mean of 1.29 ± 3.13 per patient. The 3D regimen, as well as glecaprevir/pibrentasvir and sofosbuvir/velpatasvir/voxilaprevir, presented the highest drug-drug interaction indexes.

**Conclusion:**

Comorbidities and comedications are common in patients with hepatitis C, as are drug-drug interactions. Even when second generation drugs are used, the occurrence of drug-drug interactions still presents a significant risk.

## Introduction

Chronic hepatitis C is an insidious disease that can progress to cirrhosis [1]. The goal of treatment is a sustained virological response (SVR), which with the current use of interferon-free direct-acting antivirals (DAAs) occurs in more than 90% of patients, although it does not rule out the risk of drug-drug interactions (DDIs), especially in patients who are being treated for other comorbidities [2,3].

Patients infected with hepatitis C virus (HCV) have a high prevalence of comorbidities and can be treated with several drugs [4]. In addition to late diagnosis, HCV patients are getting older and therefore at greater risk since the C virus itself can induce comorbidities, such as insulin resistance and diabetes. There are few studies on the prevalence of comorbidities in HCV-infected patients in Brazil, but they highlight the impact of comorbidities in the choice of treatment due to the drug-drug interactions [5]. Linking hepatitis C patients to health services is essential for monitoring the disease and seeking to achieve the World Health Organization’s goal of controlling it by 2030 [6]. Reducing the complexity of treatment is essential for effective primary care and, in this context, pangenotypic agents are likely to have a major role in achieving such a goal. To prepare and implement public HCV programs, it is important to assess the impact of DDIs since they could be a barrier to broader dissemination of treatment. This could help governments and healthcare professionals to develop plans to monitor data and minimize the effects of DDIs. No studies published on DDIs in HCV patients in Brazil include all available treatment agents. The aim of this study was to evaluate the risk of DDIs by determining the prevalence of comorbidities and comedications in HCV patients.

## Methods

### Study design

Retrospective cohort study with real and simulated data on medication interaction.

### Participants

2.433 electronic medical records of patients who underwent follow-up in a specialized service in southern Brazil between 2012 and 2017 have been analyzed, 1433 of wich met the positive HCV criteria with or without SVR.

### Variables analyzed

Age, sex, risk factors for HCV infection (alcohol consumption, drug addiction, HIV/HBV coinfection, organ transplantation, blood transfusion), total bilirubin, aspartate aminotransferase, alanine aminotransferase, platelets, creatinine, prothrombin time, albumin, glycemia, total cholesterol and its fractions, triglycerides, genotype and viral load, hepatic fibrosis (biopsy according to METAVIR staging and/or elastography) and clinical evidence of cirrhosis (clinical aspects), comorbidities, comedications, previous antiviral treatment, adverse reactions, and DDIs.

For the simulation the DDIs were evaluated using information available in the University of Liverpool database (https://www.hep-druginteractions.org) according to the following therapeutic regimens: (a) discontinued regimens: telaprevir (TVR); (b) regimens involving first-generation drugs: sofosbuvir (SOF)/simeprevir (SMV), SOF/daclatasvir (DCV), SOF/ledipasvir (LDV), SOF/velpatasvir (VEL), elbasvir (EBR)/grazoprevir, ombitasvir/paritaprevir/ritonavir (2D regimen) and 2D/dasabuvir (3D regimen); and (c) regimens involving second generation drugs: glecaprevir (GLE)/pibrentasvir (PIB) and SOF/VEL/voxilaprevir (VOX).

### Outcomes

Prevalence of comorbidities, comedications and DDIs in the real life data. Simulated DDIs prevalence with different DAA regimens.

The DDIs were classified as: (1) non-coadminister; (2) potential interaction; (3) weak potential interaction without clinical relevance; (4) no interaction expected. The drugs were grouped into therapeutic classes to assess the frequency of prescriptions.

### Statistical methods

Quantitative variables were described as mean ± standard deviation or median and interquartile range. Qualitative variables were described as absolute and relative frequency. Student’s *t*-test was used to compare means between groups. The results were tested for normality with the Shapiro-Wilk test. To verify possible associations, the chi-squared test with adjusted residual analyses was applied. To compare interaction severity according to the treatment regimen, matched nonparametric Friedman tests were used. The significance level was set at 5%, and the data were analyzed in SPSS^®^ 18.0.

### Ethical aspect

This study was approved by the Hospital de Clínicas de Porto Alegre Ethics Committee (n. 160657), and was registered on line (www.saude.gov.br/plataformabrasil: CAAE Ref. n°. 62487316.5.0000.5327). The study was conducted according to the Strobe Guidelines and the Ethical Standards of the Declaration of Helsinki. The researchers signed a commitment to use the data, ensuring the anonymity and confidentiality of the information.

## Results

**Fig 1:**
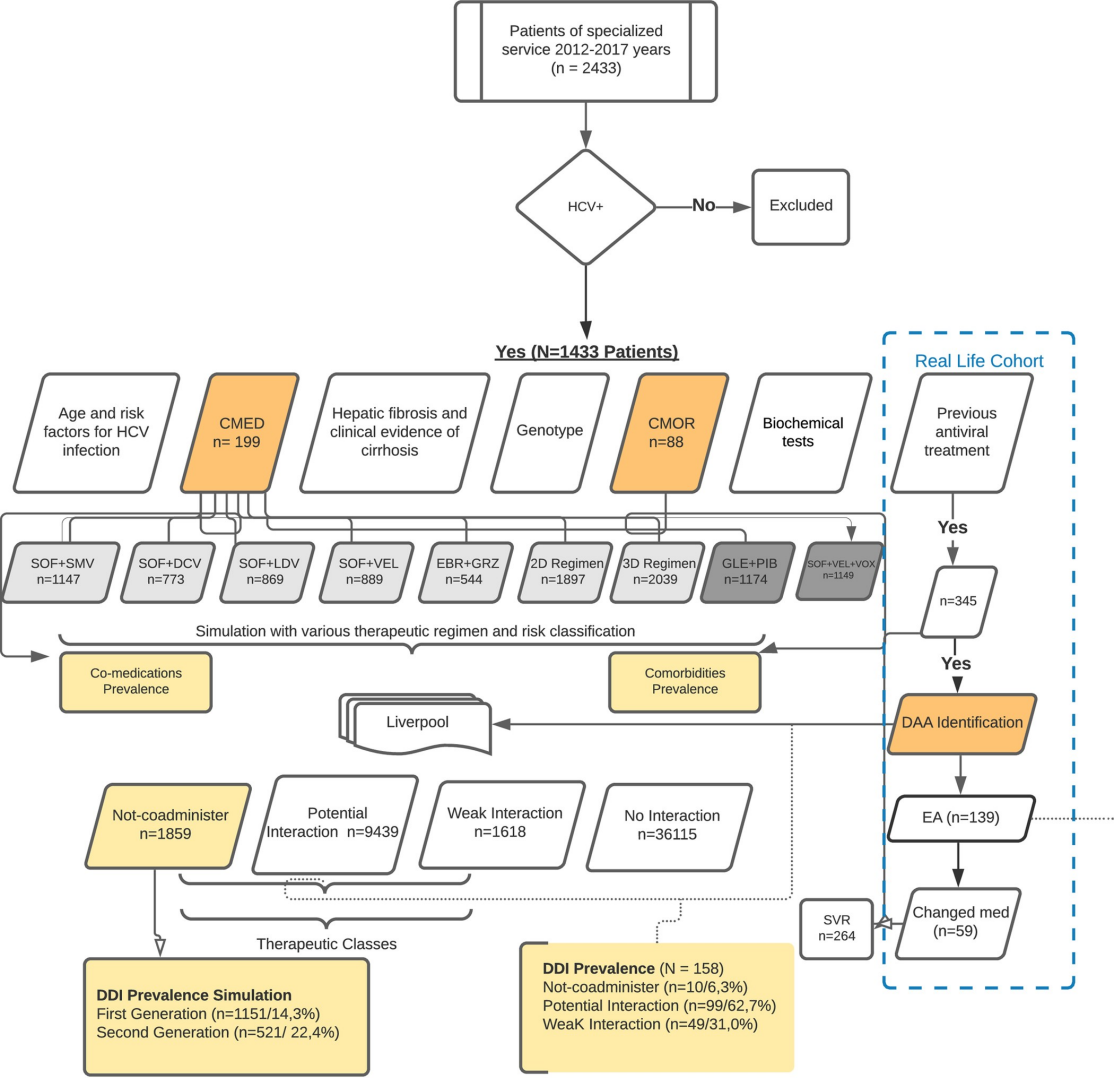
Flow diagram. Legend: HCV+ = hepatitis C viral positive; CMED = co—medications; CMOR = comorbidities; SVR = sustained virological response; Light grey box = first-generation pangenotypic DAAs; SOF+SMV = sofosbuvir and simeprevir; SOF+DCV = sofosbuvir and daclatasvir; SOF+LDV = sofosbuvir and ledipasvir; SOF+VEL: sofosbuvir and velpatasvir; EBR+GZR = elbasvir and grazoprevir; 2 D Regimen = OBV/PTV/r = ombitasvir, paritaprevir and ritonavir (r); 3D Regimen = 2D+DSV = ombitasvir/paritaprevir/r/dasabuvir; Dark grey box: second generation DAAs; GLE+PIB: glecaprevir/pibrentasvir; SOF+VEL+VOX = sofosbuvir/velpatasvir/voxilaprevir; DAA = direct-acting antivirals; EA = adverse events; med = medications; Dark orange box = main variables; Light orange box = outcomes.

We evaluated the medical records of 1433 patients diagnosed with HCV, based on records of 2433 patients of a specialized service. The comorbidities and comedications were arranged in tables to analyze interaction frequency. The Flow Diagram (Fig 1) presents the interactions data collected.

The population’s mean age was 51.7 ± 10.7 years, and 50.6% were female. The distribution of genotypes was GT1 = 732 (51.1%), GT2 = 84 (5.86%), GT3 = 510 (35.6%) and mixed genotype = 5 (0.28%). Among the risk factors for HCV infection, 342 (24.1%) patients reported alcohol abuse and 189 (13.3%) reported injected drug use. There were 178 (12.5%) transplanted patients. HIV and HBV coinfection was present in 124 (8.7%) and 19 (1.3%) of the patients, respectively.

To estimate fibrosis according to the METAVIR scale, a biopsy was used in 452 (49.6%) patients and elastography was used in 459 (50.4%). Regarding fibrosis, 100 (11.0%) patients had F0, 256 (28.1%) had F1, 169 (18.6%) had F2, and 182 (20.0%) had F3. Cirrhosis was present in 723 (50.4%) of the patients. Hepatocellular carcinoma (HCC) was identified in 199 (13.9%) patients.

Two or more comorbidities occurred in 681 patients (47.5%), and in 41 (2.86%) patients there were 5 to 7 comorbidities. Patients under 30 years of age (n = 48, 3.3%) had a median [IQR] of 1.00 [0.00–2.00] comorbidities, whereas patients over 65 years of age (n = 143, 4.5%) had a median [IQR] of 2.00 [1.00–3.00] comorbidities. There was a positive correlation between comorbidities and age group (Spearman’s correlation: rS = 0.132, p≤0.0001). The median [P25–P75] number of comorbidities was increased in the over 65 years group in relation to the <30 years and 30–65 years groups (Kruskal-Wallis with Dunn post hoc test, p≤0.0001). The main comorbidities are listed in Table 1.

**Table 1:** General description of the main comorbidities.

Comorbidities	n (%)
Arterial hypertension	436 (30.4)
Diabetes mellitus	352 (24.6)
Hypothyroidism/Hyperthyroidism	112 (7.8)
Neuropsychiatric Disorders	155 (10.8)
Depression	130 (9.1)
Bipolar Disorder	17 (1.19)
Schizophrenia	5 (0.35)
Alzheimer’s disease	1 (0.06)
Parkinson’s disease	1 (0.06)

Legend: Total comorbidities = 88.

**Fig 2:**
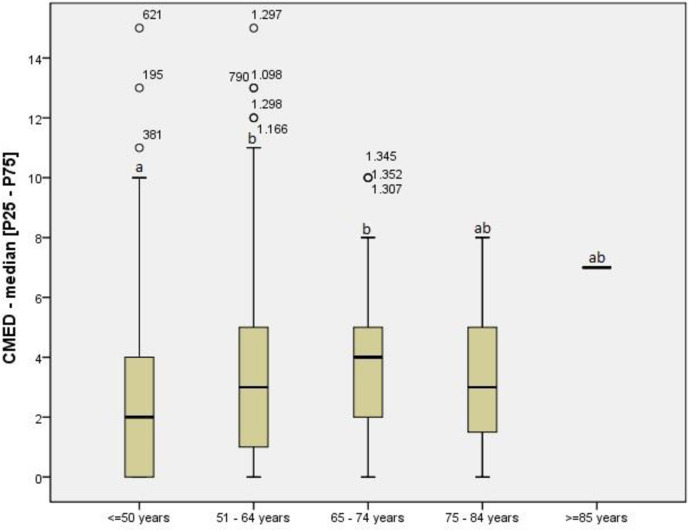
Comedications according to age group. Legend: CMED: comedications; P25-P75: interquartile range (percentiles 25th - 75th); Kruskal-Wallis test (Dunn post hoc): p≤0.0001. Significance set at 5% for all analysis. Different letters (ab) indicate Statistical significance.

A total of 199 drugs were included, the most frequent of which were: omeprazole [n = 397/27.7%], propranolol [n = 265/18.5%], furosemide [n = 187/13.0%], metformin [n = 179/12.5%], and insulin [n = 151/10.5%)]. Regarding polypharmacy, 12 patients (0.84%) used more than 10 medications concomitantly, 263 (18.35%) used from six to ten medications concomitantly and 843 (58.3%) used from 3 to 5 medications concomitantly. The number of mean comedications was 3.16±2.67 per patient. Stratifying the analysis among patients with systemic arterial hypertension (SAH), depression, and hypothyroidism/hyperthyroidism, the use of ≥10 medications occurred in 16 (3.7%), 1 (0.8%) and 2 (1.8%), respectively. The percentage was higher among diabetes mellitus (DM) patients: 5.1% used ≥10 medications. There was also a positive correlation between comedications and age group (Spearman’s correlation: rS = 0.204, p≤0.0001). The median [P25–P75] number of comedications was higher in the 51–64 and 65–74 age groups than in patients <50 years of age (Kruskal-Wallis with Dunn post hoc test, p≤0.0001), as shown in Fig 2.

DAAs were prescribed for 345 (24%) patients who represented 158 DDIs. The DDIs were distributed in categories according to the DAA regimen and generation of drug, as shown in Table 2. The SVR rate with DAAs was 76.5% (n = 264). Among the patients treated with DAAs, 139 (41%) had adverse reactions and 59 (42%) changed medication due to the adverse reaction. In the patients with DAA treatment, 177 (51,3%) were female and most of them had advanced fibrosis F3 = 80 (31,2%) and F4 = 90 (35,2%), The distribution of genotypes was GT1 = 209 (60.9%), GT2 = 12 (3.5%), GT3 = 121 (35.3%) and mixed genotype = 1 (0.3%). There were 77 (22.3%) transplanted patients and HIV was present in 19 (5,5%).

**Table 2:** Interactions with DAAs in the real life cohort.

DAA Treatment	Type of Interaction (N = 158) N (%) n/N [95%CI]		
	Non coadminister 10 (6.3) 0.06 [0.03–0.113]	Potential Interaction 99 (62.3) 0.63 [0.55–0.70]	Weak Interaction* 49 (31.0) 0.31–0.39]
**SOF_DCV_RBV** (patients n = 221/DDIs n = 106)	8 (80.0) 0.80 [0.44–0.97] SOF (n = 4)/DCV (n = 4)	54 (54.5) 0.54 [0,44–0.65] SOF (n = 0)/DCV (n = 54)	44 (89.8) 0.89 [0.77–0.97] SOF (n = 8)/DCV (n = 36)
**SOF_ RBV** (patients n = 12/DDIs n = 0)	0	0	0
**SOF_RBV _PEG** (patients n = 21/DDIs n = 0)	0	0	0
**SOF_SMV** (patients n = 61/DDIs n = 36)	1 (10.0) 0.10 [0.00–0.44] SOF (n = 1)/SMV (n = 0)	34 (34.3) 0,34 [0.25–0.44] SOF (n = 0)/SMV (n = 34)	1 (2.0) 0.02 [0,00–0.10] SOF (n = 1)/SMV (n = 0)
**TVR_PEG_RBV** (patients n = 1/DDIs n = 6)	1 (10.0) 0.10 [0.00–0.44] TVR (n = 1)	5 (5.0) 0.05 [0,02–0.11] TVR (n = 5)	0
**SOF_DCV_RBV_TPV** (patients n = 1/DDIs n = 1)	0	1 (1.0) 0.01 [0.00–0,01] TPV (n = 1)	0
**SOF_DCV** (patients n = 26/DDIs n = 9)	0	5 (5.0) 0.05 [0.02–0.11] DCV (n = 5)	4 (8.2) 0.08 [0.02–0.20] SOF (n = 0)/DCV (n = 4)
**ABT-450/r-OBV** (patients n = 1)/DDI not Analyzed	Not Analyzed	Not Analyzed	Not Analyzed

Legend: * Interaction without clinical relevance; SOF = Sofosbuvir; DCV = Daclatasvir; RBV = Ribavirin; SMV = Simeprevir; TVR = Telaprevir; PEG = Peguilado; OBV = Ombitasvir; ABT-450/r-OBV = Interferon free regim/ombitasvir and dasabuvir with ribavirin.

Table 3 shows the DDIs simulated data which were distributed in categories according to DAA regimen and generation of drug.

**Table 3:** Distribution of the interactions found according to the scheme DAAs.

		Type of Interaction (N = 49031) N (%) n/N [95%CI]			
**Generation**	**DAA**	**Not coadminister** 1859 (3.8) 0.038 [0.036–0.040]	**Potential Interaction** 9439 (19.2) 0,192 [0.189–0.196]	Interaction Weak* 1618 (3.2) 0.33 [0.031–0.035]	**No Interaction** 36115 (73.7) 0.737 [0.733–0.740]
**Abandoned**	**TVR**	187 (10.1) 0.101 [0.087–0.115]	2318 (24.5) 0,246 [0.237–0.254]	36 (2.2) 0.022 [0.016–0.031]	1923 (5.3) 0.053 [0.051–0.056]
**First Generation**	**SOF**	40 (2.1) 0.021 [0.015–0.029]	9 (0.1) 0,001 [0.000–0.002]	57 (3.5) 0.035 [0.027–0,045]	4358 (12.1) 0.121 [0.117–0.124]
	**SMV**	182 (9.7) 0.098 [0.085–0.112]	814 (8.6) 0.086 [0.081–0.092]	45 (2.8) 0.028 [0.020–0.037]	3423 (9.5) 0.095 [0.092–0.098]
	**DCV**	47 (2.5) 0.025 [0.019–0.033]	383 (4.1) 0.041 [0.037–0.45]	237 (14.6) 0.146 [0.130–0.165]	3764 (10.4) 0.104 [0.101–0.107]
	**SOF/LDV**	45 (2.4) 0.024 [0.018–0.032]	784 (8.3) 0.083 [0.078–0.089]	40 (2.5) 0.025 [0.018–0.033]	3595 (9.9) 0.099 [0.096–0.103]
	**2D**	293 (15.7) 0.158 [0.141–0.175]	1094 (11.5) 0.116 [0.109–0.122]	510 (31.5) 0.315 [0.293–0.338]	2567 (7.1) 0.071 [0.068–0.074]
	**3D**	296 (15.9) 0.159 [0.143–0.177]	1227 (12.9) 0.130 [0.123–0.137]	516 (31.9) 0.319 [0.296–0.342]	2425 (6.7) 0.067 [0.065–0.070]
	**VEL/SOF**	77 (4.1) 0.041 [0.033–0.051]	785 (8.3) 0.083 [0.078–0.089]	27 (1.7) 0.017 [0.011–0.024]	3575 (9.9) 0.099 [0.096–0.102]
	EBR/GZR	171 (9.2) 0.092 [0.079–0.106]	366 (3.9) 0.038 [0.035–0.043]	7 (0.4) 0.004 [0.002–0.009]	3884 (10.7) 0.107 [0.104–0.111]
**Second**	**GLE/PIB**	**255 (13.7) 0.137 [0.122–0.154]**	806 (8.5) 0.085 [0.080–0.091]	113 (6.9) 0.070 [0.058–0.083]	3286 (9.1) 0.091 [0.088–0.094]
**Generation**	**SOF/VEL/VOX**	**266 (14.3) 0.143 [0.127–0.160]**	853 (9.0) 0.090 [0.085–0.096]	30 (1.8) 0.018 [0.012–0.026]	3315 (9.2) 0.092 [0.089–0.095]

Legend: Total number of interactions = 49,031;

*Interactions without clinical relevance; DAA = direct-acting antiviral; TVR = telaprevir; SOF = sofosbuvir; SMV = simeprevir; DCV = daclatasvir; LDV = ledipasvir; 2D = OBV/PTV/r = (ombitasvir/paritaprevir/r/ritonavir); 3D = OBV/PTV/r+DSV (ombitasvir/paritaprevir/r/dasabuvir); VEL = velpatasvir; EBR = elbasvir; GRZ = grazoprevir; GLE = glecaprevir; PIB = pibrentasvir; VOX = voxilaprevir. Numbers and categories highlighted in bold highlight the significant interaction between the reduction of severe DDI for second generation drugs compared to the others analyzed (Friedman multiple comparison, p≤0.001).

There were 1.618 potentially weak interactions (without clinical relevance) and 36.115 possible associations with no expected interaction or insufficient data for classification. A total of 9.439 potential interactions were identified, with omeprazole, propranolol, furosemide, metformin, enalapril, tacrolimus, amlodipine and simvastatin being the most prescribed and everolimus (n = 9), warfarin (n = 8), carvedilol (n = 8), tacrolimus (n = 7), amlodipine (n = 7), omeprazole (n = 5), simvastatin (n = 5), dipyrone (n = 5), digoxin (n = 5), olmesartan (n = 5). In the LED/SOF, SOF/VEL and SOF/VEL/VOX regimens, omeprazole DDIs were classified as potential, whereas in the GLE/PIB, 2D and 3D regimens they were classified as potential or weak (without clinical relevance). Contraceptives were probably underreported in the medical records, since they were described in only one patient of childbearing age (estradiol). In this case, there would be a potential interaction with the 2D or 3D regimens and a weak potential interaction with GLE/PIB.

There were no DDIs in 1,117 patients (77.9%) but, in the simulation data, this study found a median [IQR] of 7.00 [00–14.00] DDIs, with mean of 1.29 ±3.13 of the non-coadminister type for patient. The prevalence of non-coadminister DDIs was 1151 (14.3%) and 521 (22.43%) for first and second generation drugs, respectively. The most important DDIs occurred with the 3D, 2D, GLE/PIB and SOF/VEL/VOX regimens. When the non-coadminister DDIs were analyzed in the Friedman multiple comparison test, there was no difference between SOF, DCV, EBR/GRZ, SOF/LDV and VEL/SOF (p> 0.05), and their DDI prevalence was lower than the second generation drugs (p <0.001)(Table 3). There was no significant difference between the DDIs of GLE/PIB and SOF/VEL/VOX (p = 0.478).

A total of 1859 high-risk DDIs (non-coadminister type) were found, mainly with simvastatin (n = 425), lopinavir (n = 217), carbamazepine (n = 204), efavirenz (n = 168), phenobarbital (n = 120), phenytoin (n = 96), atorvastatin (n = 75), atazanavir (n = 70), cyclosporine (n = 64), domperidone (n = 56), amiodarone (n = 45), quetiapine (n = 33), budesonide (n = 16), fluconazole (n = 14) and gemfibrozil (n = 3). The main drugs that cause DDIs non coadminister in the simulated data are listed in Table 4 (Table 4).

**Table 4:** Interactions according to physiological system.

		Interaction with non-coadminister DAA											
		Antiviral Regimens DAAs by Generation											
		Discontinued	First Generation									Second Generation	
System	Main prescribed drugs (n/%)	TVR^(d)^	DCV^(p)^	SOF/DCV^(p)^	SOF/VEL^(p)^	SOF/SMV^(1)^	SMV^(1)^	SOF/LDV^(1)^	EBR/GRZ^(1)^	2 D	3 D	GLE/PIB^(2)^	SOF/VEL/VOX^(2)^
Cardiovascular	Amiodarone (5/0.35%)												
Endocrine	Simvastatin (85/5.93%)												
	Atorvastatin (15/1.04%)												
	Gemfibrozil (3/0.21%)												
Neuropsychiatric	Carbamazepine (17/1.19%)												
	Phenobarbital (10/0.70%)												
	Phenytoin (8/0.56%)												
	Quetiapine (11/0.77%)												
Respiratory	Budesonide (8/0.56%)												
	Fluticasone (2/0.14%)												
Rheumatologic	Metamizole (31/2.16%)												
	Methotrexate (4/0.28%)												
Dermatological	Dexamethasone (2/0.14%)												
Gastrointestinal	Domperidone (28/1.95%)												
Antibiotic Anti-infection Antiviral^†^	Efavirenz (28/1.95%)												
	Atazanavir (14/0.97%)												
	Lopinavir (31/2.16%)												
	Nevirapine (4/0.27%)												
	Saquinavir (1/0.07%)												
	Rifampicin (5/0.35%)												
	Clarithromycin (2/0.14%)												
	Fluconazole (7/0.49%)												
Immunosuppressants	Tacrolimus (117/8.16%)												
	Cyclosporine (16/1.12%)												

Legend: DAA in disuse: TVR: Telaprevir; (p) first-generation pangenotypic DAAs: DCV: Daclatasvir; SOF/DCV: Sofosbuvir and daclatasvir; SOF/VEL: Sofosbuvir and velpatasvir; (1) first-generation DAAs: SOF/SMV: Sofosbuvir and simeprevir; SMV: Simeprevir; SOF/LDV: Sofosbuvir and ledipasvir; GRZ/EBR: Grazoprevir and elbasvir; 2 D = OBV/PTV/r = ombitasvir, paritaprevir and ritonavir; 3D = 2D+DSV = ombitasvir/paritaprevir/r/dasabuvir; (2): Second generation DAAs: GLE+PIB: Glecaprevir/pibrentasvir; SOF/VEL/VOX: Sofosbuvir/velpatasvir/voxilaprevir; Red = non-coadminister interaction; Green = no non-coadminister interaction;

^†^ mixed therapeutic class.

Of the 199 drugs used by the patients, 25 (12.5%) are not included in the Liverpool database. The consumption of teas, supplements and herbal products was not analyzed.

## Discussion

This study simulated DDI occurrence in the antiviral regimens of patients with HCV and, according to our knowledge, this is the first such analysis to include second generation drugs.

A high rate of comorbidities and comedications was found, which indicated a significant number of DDIs. The DDIs non coadminister in the patients in treatment with SOF_DCV_RBV were represented by carbamazepine and fenobarbital, which are potent inducers of P-gp and may significantly decrease sofosbuvir concentrations. This may result in loss of efficacy and potential virological failure however patients have achieved SVR. One DDI non coadminister in the patient in treatment with SOF_SMV was represented by amiodarone. Although this mechanism of the effect is unknown, coadministration of amiodarone and sofosbuvir combined with simeprevir may result in serious symptomatic bradycardia and is not recommended. In this case, the patient did not show EA and have achieved SVR.

In the simulated data DDIs occurred more frequently among first-generation 2D and 3D regimens, which are falling goingout of use, but also with the second generation GLE/PIB and SOF/VEL/VOX regimens. This demonstrates that the introduction of these pangenotypic and co-formulated DAAs is unlikely to reduce DDIs, which could negatively impact the advance of hepatitis C treatment, since DDIs are an important barrier to prescription in primary health care [7].

The mean age of the patients was similar to previous HCV studies [3,8], and the genotypic distribution agreed with previous findings [9,10]. The prevalence of genotype 3 was 35.6%, slightly lower than that reported in other studies in southern Brazil [11]. The number of patients with advanced fibrosis was over 70%, which shows the severity of the cases. Cirrhotics should be considered to have impaired cytochrome P450, which indicates a higher risk of toxicity in cases of DDI [12,13]. The most prescribed antiviral regimen was SOF/DCV/RBV, which was the leading treatment in Brazil at the time [14] This could explain the lower SVR rate found in this study compared to previous publications [9,12]. However, a Brazilian study using a 3D regimen in F3-F4 patients found an SVR rate over 95% (Mario Pessoa et al., *Ann Hepatol* in press). Thus, the retrospective character of our study could have produced an additional limitation.

Regarding comorbidities, about half of the population had two or more associated diseases, and this was more frequent in patients over 65 years of age than in other studies [10,15]. When prescribing drugs to older people, it is necessary to consider not only age-related pharmacokinetic and pharmacodynamic changes, but altered body functions and the risk of DDIs in real-world practice. The most frequent comorbidities were SHA, DM and depression, as described in other studies [9,12]. Among these comorbidities, DM is causally related to HCV [16], and it is speculated that depression may be associated with viral replication in the central nervous system [15]. On the other hand, renal manifestations of HCV are frequent and could be associated with hypertension [17]. Increased comorbidities and their chronic character contribute to polypharmacy, which is considered the concomitant use of five or more medications. Moreover, self-medication may occur in some cases.

Proton pump inhibitors were the most common comedication in the sample, followed by propranolol, furosemide and metformin, all of which were commonly used in this population. Omeprazole is an over-the-counter medication that is often used indiscriminately, which could lead to potential interaction with DAAs. Proton pump inhibitors were used by 27.7% of the patients in this sample, which replicated the findings of U.S. studies [8,13]. These studies did not analyze interactions with second-generation DAAs, but this seems important, since these drugs have a potential interaction with SOF/VEL/VOX and weak potential interaction with GLE/PIB. Omeprazole doses higher than 20mg/day are not recommended because they interfere with the absorption of VEL [7]. Omeprazole also interferes with the absorption of 3D and SOF/LDV, although no loss was found with SVR [18,19].

The present study analyzed DDIs with all currently available DAAs on the market, finding twice the rate of non-coadminister interactions (16.11% versus 8.8%) as U.S. studies. The most important DDIs involved the 2D, 3D and second generation regimes. Kondilli et al [18]. evaluated first-generation DAAs and found that the 3D regimen had the greatest possibility of interactions. Table 5 shows the results of published studies on this outcome. Rocero et al. (2018) [20] simulated interactions between first and second-generation DAAs and psychotropic drugs, finding that SOF-based regimens have fewer DDIs than those which include protease inhibitors [21].

**Table 5:** Published studies on drug interactions with direct-acting antivirals in hepatitis C patients.

Author. Year	N	DAA Generation	Design	% Comorbidities	% Comedications	% DDI high risk (n)	Conclusion
Höner Zu Siederdissen et al. 2016 [8]	261	First -generation	Retrospective	NA	Mean 2 (0–15)/patient	0.4% (1)	There are a significant number of DDIs with DAAs
Soriano et al. 2017 [21]	N/A	First -generation	Review	N/A	N/A	N/A	Even with pangenotypic DAAs, DDIs remain a challenge
Kondilli et al. 2018 [18]	449	First -generation	Prospective*	N/A	More than 3 drugs reported in 22% of the patients (19)	N/A	30–44% of patients on DAAs had clinically significant DDIs
Hudson et al. 2017 [9]	6278	N/A	Retrospective	Depression: 26.1%; DM 11.3%; HIV 5.0%	Anti-diabetics 9.3% and Statins 4.9%; Age>60 years: Anti-diabetics 18.8% Statins 11.7%.	N/A	Comorbidities and polypharmacy have a high risk of DDIs.
Mazzarelli et al. 2018 [12]	113	First–generation	Retrospective	83% were 65–74 years old and 88% were >74 years old.	Patients ≥75 years >2 comedications (84% vs 62%. p = 0.02); cirrhosis patients: uses ≥5 pills/day (56% vs 39%. p = .008).	Patients ≥75 years >frequency DDIs; 2 DDIs (80% vs. 36%, p = 0.001)	Second generation DAAs are simpler and safer for elderly patients
Ottman et al. 2018 [13]	560	First -generation	Retrospective	N/A	PPIs: 20.4% (113); Statins: 15.7% (87); Antidepressants: 7.8% (43)	8.8% (49) with severe DDIs; 3D - 16.6%;	The 3D regime had fewer severe DDIs;
Roncero et al. 2018 [20]	N/A	First and second generation	Pharmacology review	N/A	Psychoactive drugs	N/A	DAAs with SOF have fewer DDIs than PI. GLE/PIB and GRZ/ELB. has fewer DDIs than 3D.

Legend: HCV: Chronic hepatitis C; DAA: Direct-acting antivirals; N: Sample size; comorbidities: Comorbidity; comedications: Comedication. DDI: Drug-drug interaction; n: Number; PPI: Proton pump inhibitors; PI: Protease inhibitors; GLE: Glecaprevir; PIB: Pibrentasvir; GRZ: Grazoprevir; ELB: Elbasvir; 3D: Ombitasvir/paritaprevir/r/dasabuvir; DM: Diabetes mellitus; SOF, sofosbuvir.

In this study, 1859 possibilities of high-risk (non-coadminister) DDIs were found, mainly with simvastatin (n = 425), lopinavir (n = 217), carbamazepine (n = 204), efavirenz (n = 168), phenytoin (n = 96), atorvastatin (n = 75), phenobarbital (n = 50), cyclosporine (n = 64), domperidone (n = 56), amiodarone (n = 45), quetiapine (n = 33), budesonide (n = 16), fluconazole (n = 14), and gemfibrozil (n = 3). Concerning simvastatin, important DDIs occur with the 2D and 3D regimens, as well as with newer generation drugs [8,13]. In fact, administering statins with GLE/PIB or SOF/VEL/VOX increases the risk of myopathy and rhabdomyolysis. Antiretroviral DDIs are extensively discussed in the literature [17]. Lopinavir increases the systemic concentration of GLE/PIB, whereas efavirenz may significantly decrease its plasma concentration. Amiodarone, a calcium channel inhibitor, should not be used with SOF/VEL/VOX due to the risk of bradycardia, nor should it be used with the 2D or 3D regimens.

Previous studies have found a mean of 1.85 DDIs per patient (range 1–9), with 80.3% of the patients having at least one DDI and 76% having a potentially critical interaction. However, these studies did not consider second generation regimens, which, according to our results, represent 5.62 DDIs per patient (n = 8052) when only serious and potential DDIs are considered.

The limitations of this study are its retrospective nature, which precluded interviewing patients to verify the exact doses of comedications used and establishing causality between HCV exposure and disease. Because there was no interview, we could not assess the use herbal products and other common medications, like estrogen. The analysis simulated the possibility of interactions between drugs and was limited to the qualitative character of the DDIs, rather than dose-dependent effects. Thus, only non-coadminister DDIs were discussed. The SVR rate was not assessed according to comorbidities and comedications since the DDIs were simulated. Furthermore, 25 comedications were not found in the Liverpool database, which demonstrates the individuality of each population and the importance of adjusting treatment for regional differences.

In conclusion, comorbidities and comedications are common in patients with HCV and DDIs often occur. Even with second generation drugs, DDIs continue to present a significant risk.

## Supporting information

S1 Table Co-medications of the patients with HCV. (DOCX)

## Abbreviations

HCV: hepatitis C virus
DDI: drug interactions
DAA: direct-acting antivirals
TVR: telaprevir
SMV: simeprevir
PTV: paritaprevir
GRZ: grazoprevir
VOX: voxilaprevir
GLE: glecaprevir
DCV: daclatasvir
LDV: ledipasvir
OBV: ombitasvir
EBR: elbasvir
VEL: velpatasvir
PIB: pibrentasvir
SOF: sofosbuvir
DSV: dasabuvir
RBV: ribavirin
2D: veruprevir/ritonavir,ombitasvir
3D: veruprevir/ritonavir,ombitasvir/dasabuvir
SVR: sustained virological response
SAH: systemic arterial hypertension
DM: diabetes mellitus
SD: Standard deviation
