# Correction: Direct antiviral agents for hepatitis C and drug interaction risk: A retrospective cohort study with real and simulated data on medication interaction, prevalence of comorbidities and comedications

**DOI:** 10.1371/journal.pone.0269049

**Published:** 2022-05-23

**Authors:** Raquel Boff da Costa, Marisa Boff Costa, Larisse Longo, Daniela Elisa Miotto, Gustavo Hirata Dellavia, Matheus Trucollo Michalczuk, Mario Alvares-da-Silva

The seventh author’s name is spelled incorrectly and his ORCID iD is missing. The correct name is: Mario Alvares-da-Silva. Please see the author’s ORCID iD here:

Author Mario Alvares-da-Silva’s ORCID iD is: 0000-0002-5001-246X (https://orcid.org/0000-0002-5001-246X).
