# Supplementary material for: Direct antiviral agents for hepatitis C and drug interaction risk: A retrospective cohort study with real and simulated data on medication interaction, prevalence of comorbidities and comedications
Source: PLoS One. 2021 Feb 12;16(2):e0245767. doi: 10.1371/journal.pone.0245767 (PMC7880426; doi:10.1371/journal.pone.0245767)
Supplement: S1 Table — (DOCX) [file pone.0245767.s001.docx]

| Co-medications of the patients with HCV | | | |
| --- | --- | --- | --- |
| aspirin | cholecalciferol (vitamin D) | isossorbide mononitrate | periciazine |
| abacavir | chlorpromazine | iron supplements | phenytoin |
| acarbose | chlortalidone | lactulose | phenobarbital |
| acetilcisteína | clozapine | lamivudine | piroxicam |
| aciclovir | codeine | lamotrigine | pramipexole |
| folic acid | colestyramine | leflunomida | pravastatin |
| ursodeoxycholic acid | dapagliflozin | levodopa | prednisone |
| adalimumab | darunavir | levomepromazine | pregabalin |
| agomelatine | dexamethasone | levothyroxine | progesterona |
| albendazole | desvenlafaxine | linagliptin | promethazine |
| alendronic acid | dexchlorfeniramine | lisinopril | propranolol |
| allopurinol | diazepam | lithium | quetiapine |
| alprazolam | didanosine | loperamide | raltegravir |
| amantadine | digoxin | lopinavir | ramipril |
| amiloride | domperidone | loratadine | ranitidine |
| amiodarone | doxazosin | lorazepam | ribavirin |
| amitriptyline | efavirenz | losartana | riboflavin |
| amphotericin B | enalapril | metamizole | rifampicin |
| amlodipine | entecavir | methadone | risperidone |
| atazanavir | eritropoetina | metformin | ritonavir |
| atenolol | escitalopram | methyldopa | salbutamol |
| atorvastatin | estradiol | metilphenidate | saquinavir |
| azathioprine | etanercept | metimazol | scopolamine |
| azithromycin | everolimus | metoclorpramide | sertraline |
| baclofen | fenoterol | metoprolol | sevelamer |
| beclometasone | filgrastim | methotrexate | sildenafil |
| bezafibrate | finasteride | metronidazole | simvastatin |
| bicalutamida | fluconazole | mirtazapine | sodium diclofenac |
| biperideno | flunarizina | mycophenolate | sorafenib |
| bisacodyl | fluoxetine | morphine | sotalol |
| bromazepam | fluticasone | mupirocin | spironolactone |
| bromoprida | formoterol | naltrexone | sulpiride |
| budesonide | furosemide | nebivolol | tacrolimus |
| bupropion | gabapentin | nevirapine | tenofovir |
| cafeína (trimetilxantine) | gemfibrozil | nifedipine | terlipressin |
| captopril | glibenclamide | nimesulida | tiamazol |
| carbamazepine | glimepiride | nitrofurantoin | tiotropium |
| calcium carbimide | glucosamida | norethisterone | tramadol |
| carisoprodol | goserelin | norfloxacin | trazodone |
| carvedilol | haloperidol | nortriptyline | trimethoprim/ sulfamethoxazole |
| cefepima | heparin | olmesartan | valproate |
| cyclobenzaprine | hydralazine | ornithine aspartat | valsartan |
| cyclosporine | hydroclorotiazide | omeprazole | venlafaxine |
| cilostazol | hidroxyzine | ondansetron | verapamil |
| ciprofibrato | ibuprofen | orlistat | vildagliptin |
| citalopram | imipramine | oxibutinina | vigabatrin |
| clarithromycin | indometacin | pantoprazole | warfarin |
| clonazepam | insulin | paracetamol | zidovudine |
| clonidine | ipatrópio | paroxetina | zolpidem |
| clopidogrel | isoniazid | phenprocoucom |  |
| Legend: Green box = ausent in Liverpool Hep Drugs (n=26); Orange box = comedication on life treatment | | |  |
